# Stop turning a blind eye!

**DOI:** 10.1186/s42155-021-00261-3

**Published:** 2021-10-07

**Authors:** Jim A. Reekers

**Affiliations:** Emeritus Professor of Interventional Radiology, Amsterdam UMC, University of Amsterdam, Meibergdreef 9, Amsterdam, The Netherlands

The endovascular revolution in the treatment of peripheral artery disease (PAD) started in 1964 with Charles Dotter, and due to great technical and device-related achievements, especially in the last 15 years, we now have a wide range of new treatment options at our disposal. Refurbishments of the existing technologies are still to be expected, but a fundamental or game-changing development is however not at the horizon, yet. We are stuck in our old thinking about the etiology of Chronic limb-threatening ischemia (CLTI), where flow and restoring flow and pressure measurements are still dominant in our thinking. And we have a blind eye for the facts. If we want to move forward and if we want to get out of this dead-end street we have to reset our current thinking about CLTI. The uncomfortable truth, after all the years of innovation, is that the fate of patients with CLTI is still poor. They continue to face a 15%–30% amputation and a 30% mortality risk at 1 year depending on the level of disease severity (Duff et al. 2019). CLTI is probably not only a vascular inflow problem but also a more general expression of biological aging. Regarding the amputation risk, there are strong indications that our new endovascular revascularization techniques reduce the number of reinterventions but unfortunately not always the amputation risk (Duff et al. 2019). We also have to acknowledge that the 30-day readmission rate in patient-treated endovascular or surgical procedures for CLTI is still disturbing high, 15%–25% (Kolte et al. 2017). Most endovascular trials investigating PAD treatments are flawed by using proxy endpoints for success, patient selection bias and ignoring hard clinical endpoints, like amputation free survival, and might therefore show an over-optimistic view. The most unpredictable and frustrating observation we encounter is that a good technical revascularization result, currently still measured as an angiographic improvement of flow, does not always match with the poor real live clinical follow-up. We have acknowledged for a long time that there is something like microvascular problems and neuropathy, especially in diabetic patients, but we have never developed a research pathway to objectively diagnose this. Moreover, microcirculation and diabetic neuropathy have become an excuse diagnosis to explain away treatment failure. However, cardiologists have made the same observations more than a decade ago, and after close observation and rethinking the mechanism of tissue ischaemia, they adopted the idea of Ischaemia and Non-Occluded Coronary Arteries (INOCA) (Jespersen et al. 2012). Currently 40–60% of all patients with a coronary syndrome are (also) diagnosed as INOCA (Bairey Merz et al. 2017). Occlusive disease by atherosclerosis and INOCA can overlap in the same patient, which means that after a successful coronary revascularization there can still be remaining symptoms of pericardial ischaemia, leading to major cardiac events (Jespersen et al. 2012; Bairey Merz et al. 2017). The accepted etiology, which serves as the hypothesis for INOCA, is microvascular dysfunction, leading to shortage of the oxygen in the tissue cells. Although many specific pathophysiological pathways of INOCA are still unknown, there seems to be a great overlap with the well-known causes for atherosclerosis. It does not seem very outlandish to presume that similar pathology might also be recognized in the peripheral vessels, but currently no steps have been undertaken to investigate and identify Ischaemia in Non-Occlusive Peripheral Arteries (INOPA). The often-heard argument that coronary arteries are different from peripheral arteries is not supported by any science and is only a false presumption. INOPA could therefore be the explanation for amputations despite successful revascularization. The next step, after acknowledging INOPA as a new disease in PAD, is to identify and diagnose patients with this microvascular dysfunction.

In cardiology, there have been new tests developed like measurement of Coronary Flow Reserve (CFR), the index of microvascular resistance (IMR) and evaluation of angina induced by intracoronary acetylcholine infusion. Because of the movement of the heart imaging, direct tissue perfusion imaging is still a challenge. However in PAD we have the possibility to directly image total tissue perfusion with perfusion angiography, using specially developed quantitative software. Measuring change in total tissue perfusion after challenging the autoregulation of the microcirculation is a proxy parameter for the functionality of the microcirculation (Schreuder et al. 2018). It was shown that poor functionality of the sympathetic nerve, which can sometimes be an additional part of diabetic neuropathy, is a very strong parameter for early amputation in CLTI (Schreuder et al. 2018). This observation is supported by older publications that also have reported that dysfunctionality of the sympathetic nerve is a strong independent predictor for early amputation, an observation that has unfortunately not attained much attention. This technique of direct imaging of total tissue perfusion opens up a whole new realm of possibilities for research into INOPA, avoiding some of the cumbersome cardiology proxy endpoint tests, and could set our research agenda for the next decade. To move CLTI treatment to a higher level, we should stop turning a blind eye to INOPA and recognize that this might also play an important role in CLTI. All our current parameters to measure CLTI, like pressure and flow, were introduced by vascular surgery more than 25 years ago. We, as imagers have the option to investigate and to develop new imaging technologies that are better suited for the challenges of today. We should stop thinking of CLTI as a reduction of blood flow to the tissue due to occlusive disease and adapt a more physiological definition: A shortage of oxygen in the tissue due to an imbalance between oxygen demand and availability of tissue oxygen, by any cause. More understanding of CLTI and INOPA will help us with better patient selection, which has always been the road to better treatment results.

## Data Availability

Not applicable.
